# Research Progress of Aluminum Alloy Welding/Plastic Deformation Composite Forming Technology in Achieving High-Strength Joints

**DOI:** 10.3390/ma16247672

**Published:** 2023-12-15

**Authors:** Gang Song, Zejie Wang, Xiaoyu Fan, Liming Liu

**Affiliations:** Key Laboratory of Liaoning Advanced Welding and Joining Technology, School of Material Science and Engineering, Dalian University of Technology, Dalian 116024, China; zejie_wang@163.com (Z.W.); fxy_0718_0612@163.com (X.F.); liulm@dlut.edu.cn (L.L.)

**Keywords:** aluminum alloy, plastic deformation strengthening, plastic deformation welding, mechanical property

## Abstract

Fusion welding causes joint deterioration when joining aluminum alloys, which limits the use of aluminum alloy components in high-end equipment. This paper focuses on an overview of how to achieve high-strength aluminum alloy welded joints using welding/plastic deformation composite forming technology. The current technology is summarized into two categories: plastic deformation welding and plastic deformation strengthening. Plastic deformation welding includes friction stir welding, friction welding, diffusion welding, superplastic solid-state welding, explosive welding, and electromagnetic pulse welding. Plastic deformation strengthening refers to the application of plastic deformation to the weld seam or heat-affected zone, or even the whole joint, after welding or during welding, including physical surface modification and large-scale plastic deformation technology. Important processing parameters of plastic deformation welding and their effects on weld quality are discussed, and the microstructure is described. The effect of plastic deformation strengthening technology on the microstructure and performance evolution, including the hardness, tensile strength, fatigue property, residual stress, and hot cracking of aluminum alloy welded joints, and its evolution mechanism are systematically analyzed. Finally, this paper discusses the future development of plastic deformation strengthening technology and anticipates growing interest in this research area.

## 1. Introduction

As a green metal, aluminum (Al) alloy has been widely used in the automotive and aerospace industries due to its excellent formability, low density, and high specific strength. The manufacturing of aluminum alloy components, such as aircraft propellers, aircraft skins, vehicle frames, and car bumpers, often requires welding [1,2]. However, due to the high thermal conductivity and linear expansion coefficient of Al alloys, the traditional welding process is prone to porosity, hot cracking, low plasticity, and low joint strength, which limits the use of traditional welding methods [3].

The main strengthening mechanism of high-strength aluminum alloys, represented by 2xxx, 6xxx, and 7xxx, is precipitation hardening [4,5]. In the fusion welding process, such as gas tungsten arc welding (TIG), metal inert gas welding (MIG), laser beam welding (LBW) and cold metal transfer welding (CMT), the high-temperature thermal cycle causes the dissolution, transformation, and coarsening of the precipitation phase [6]. They weaken the hardening effect of the original precipitation phase in the matrix, resulting in aging softening of the heat-affected zone (HAZ) [7,8]. To avoid welding hot cracking, low-strength filler wires are usually employed for welding high-strength aluminum alloys, which results in lower properties for the fusion zone (FZ) [9]. In addition, high heat input leads to coarse grain and vaporization of the strengthening elements in the FZ, which reduces the content of precipitated phases and further degrades the properties of the FZ [10]. Therefore, the softening of the HAZ and the deteriorated properties of the FZ together result in the poor mechanical properties of high-strength aluminum alloy fusion welded joints. Table 1 lists the tensile properties of high-strength aluminum alloy butt joints welded by the fusion welding method, where a significant reduction in joint strength and elongation (El) occurs. The joint efficiency is the ultimate tensile strength (UTS) ratio between the joint and base metal (BM).

Great efforts have been made to improve the microstructure and mechanical properties of Al alloy welded joints. By applying irreversible plastic deformation to the metal, researchers have demonstrated that plastic processing can cause grain deformation and distortion in the deformed zone, increase dislocation density, and induce fine-grain hardening and precipitation hardening [22]. The works suggest that plastic deformation plays an important role in reducing porosity, hot cracking, and other casting defects [23,24]. In addition, it can also effectively improve the mechanical properties of metals, such as the tensile strength, microhardness, and wear resistance. Therefore, the combination of welding and plastic deformation is attractive.

Up to now, two main research approaches have been used in welding/plastic deformation hybrid forming technology. Firstly, the welding methods that directly utilize plastic deformation include friction stir welding (FSW) [25,26], friction welding (FW) [27,28], diffusion bonding (DB) [29,30], superplastic solid-state welding [31,32], explosive welding [33], and electromagnetic pulse welding (EMPW) [34]. Previous studies have shown that by optimizing process parameters, the strength of welded joints can reach more than 80% of the base metal [35,36]. Secondly, the post-welding treatment applies a certain amount of plastic deformation to the weld or the surrounding area, i.e., plastic deformation in the weld zone [37,38,39]. For example, in the case of high-strength aluminum alloy represented by the 7xxx series, some studies have shown that the joint strength is only 50% to 65% of the base metal when joined by traditional fusion welding methods [40,41]. Even with laser welding, electron beam welding, laser-arc hybrid welding, etc., the joint strength can only reach ~79% [10,42,43]. The plastic deformation in the weld zone, such as rolling, can increase the joint strength by about 24% [44].

This paper comprehensively reviews the detailed research progress and results of welding/plastic deformation composite forming technology in terms of the microstructure evolution and mechanical properties of the welded joints of Al alloy. First, this paper introduces various plastic deformation welding methods. Then, plastic deformation strengthening methods, including physical surface modification and large-scale plastic deformation technology, are also reviewed. Important processing parameters and their effects on weld quality are discussed, and the microstructure is described. The mechanical properties of welds, including the hardness, tensile strength, and fatigue strength, are also examined.

## 2. Plastic Deformation Welding

### 2.1. Friction Stir Welding

**Figure 1:**
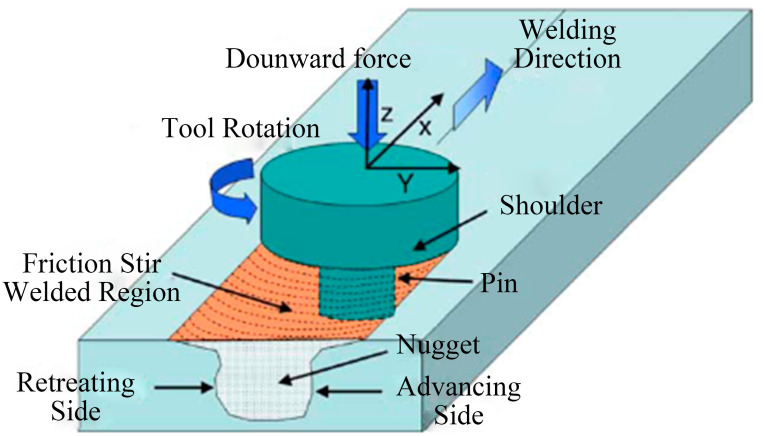
Basic working principle of FSW [45].

Friction stir welding is a solid-state joining process with relatively simple equipment. The principle of FSW is shown in Figure 1. In this joining technique, under the influence of the rotating pressure from the pin, the softened material experiences plastic flow, fills the cavity generated by the movement of the tool, and forms the weld. The weld zone comprises distinct regions, including the stir zone (SZ), the thermomechanical-affected zone (TMAZ), the heat-affected zone, and the base metal. The SZ features equiaxed grains, while those in the TMAZ and HAZ are partially recrystallized and appear less uniformly sized compared with those in the SZ.

The mechanical properties of FSW-processed joints are influenced by process parameters such as the rotation speed of the welding tool [46], welding speed, and down force. The very low rotation speed results in lower heat input and defects such as cracks and pinholes in the friction stir zone, resulting in lower tensile properties. As the speed increases, the heat input increases. An elevated temperature results in grain enlargement, which in turn leads to poorer tensile properties. As a result of the lack of bounding, the UTS decreases while the behavior of tensile strength and ductility mirror each other, resulting in a decrease in ductility [47]. A higher welding speed leads to inadequate and improper mixing of materials, and the cavity defect occurs in the SZ, which leads to stress concentration, decreases the loading area, and reduces the tensile strength [48,49]. On the other hand, the microhardness and tensile strength of the joint tend to increase as the welding speed decreases. The down force helps to maintain contact between the FSW tool and the metal surface. Decreased downforce leads to tunnel defects due to reduced heat input. Consequently, both the UTS and microhardness are reduced. Conversely, a heat input above the desired level results in worm holes and local thinning of the weld plate, which reduces UTS and ductility [50,51,52]. The following equation can calculate the heat input per unit [53]:

(1) $$Q=\frac{4}{3}\pi^{2}\alpha \mu PR^{3}\frac{\omega }{v}$$

where *Q* is the heat input per unit, *α* and *μ* are the heat input efficiency and friction coefficient, respectively, *P* is the rotating pressure, *R* is the radius of the shoulder, *ω* is the rotation speed, and *v* is the welding speed. Researchers [54,55] found that with the increase in the *ω*/*v* ratio, the joint strength and elongation initially increase, followed by a decrease. Optimal performance can only be achieved when the *ω*/*v* value is moderate.

**Figure 2:**
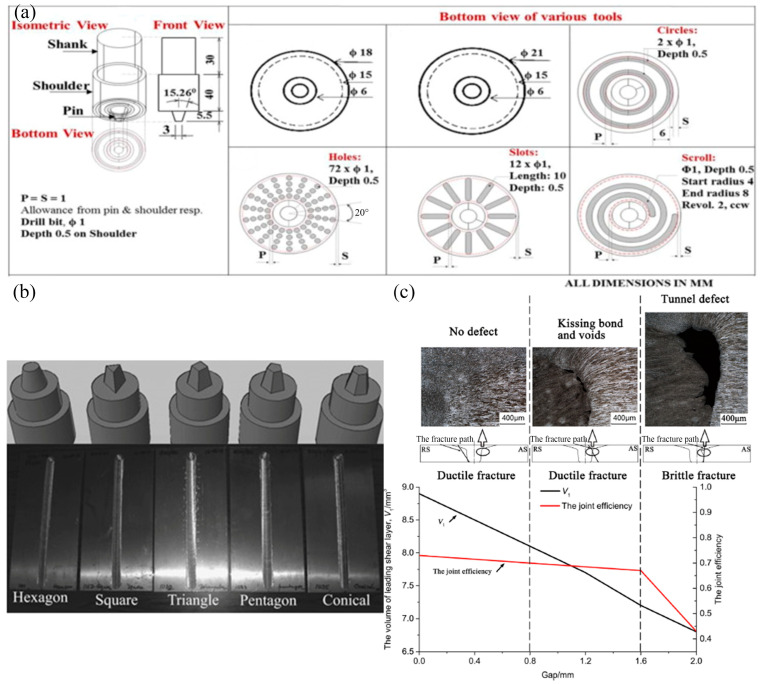
(**a**) Fabricated tool for FSW with different shoulder profiles [61]; (**b**) geometries of pin profiles and weld joints created with different pins [62]; (**c**) gap-tolerance control window for friction stir butt welding of 2A14 aluminum alloy [57].

The shape of the stirring pin and shoulder [56] and the assembly gap of the plate [57] also affect the performance of the joint, as shown in Figure 2. The shape of the stirring tool influences recrystallization and grain growth behavior by affecting the heat input, force and torque, and material flow [48,58]. The presence of a gap in the plate reduces the material availability at the interface, which affects the heat input and material flow in the vicinity of the tool pin profiles. The UTS, YS, and strain continue to decrease with increasing gap width. If the gap is too large, the material transferred by the tool will not completely fill the cavity formed by the forward movement of the pin [59,60]. Despite the large influence of process parameters, the FSW method can still obtain excellent aluminum alloy welded joints, and Table 2 shows the mechanical properties of FSW aluminum alloy butt joints.

**Figure 3:**
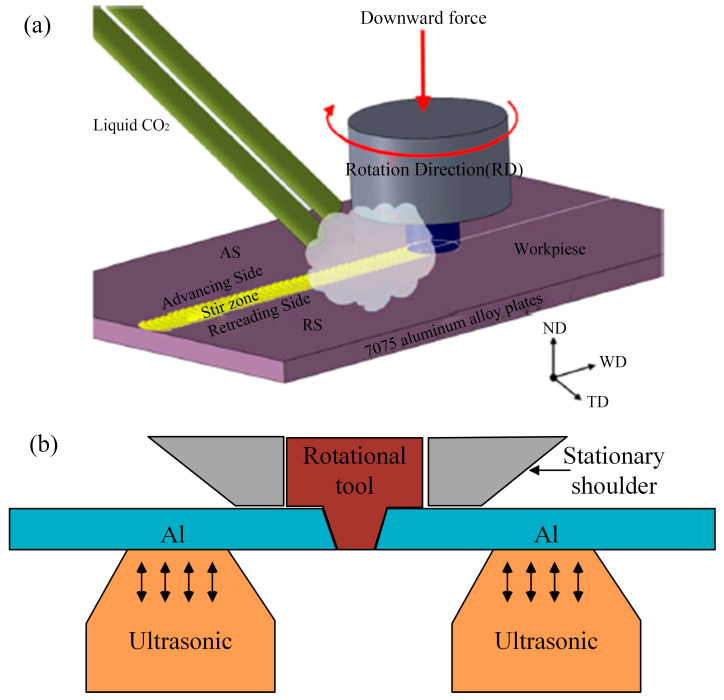
(**a**) The principle of friction stir welding assisted by carbon dioxide [78]; (**b**) schematic diagram of ultrasonic-assisted friction stir welding.

The performance of the joints can be further improved by external auxiliary means. As shown in Figure 3a, the application of forced cooling (including gas cooling and water cooling [68]) during the welding process can inhibit the dissolution of precipitated phases and the coarsening of grains by shortening the influence time of the high temperature. Compared with natural cooling, the UTS can be increased by 10% [69]. By presetting a Zn particle interlayer in the gap between the butt plates of AA6082 aluminum alloy, the FSW-processed joint can have a higher microhardness. Although the tensile strength of the joint without the interlayer is comparable, it has better fracture toughness [70,71]. It is mainly due to the formation of an Al-Zn solid solution and due to grain refinement caused by the dispersion of Zn particles [72]. In addition, the Cu interlayer can have a similar purpose [73]. As shown in Figure 3b, ultrasonic assistance plays an important role in the FSW of aluminum alloys with dissimilar metals by breaking continuous Al pieces into small Al pieces or particles, with the result that small Al pieces and their surrounding intermetallic compounds (IMCs) are dispersed in the SZ [74,75]. As the ultrasonic power increases, the tensile strength increases first and then decreases [76,77].

While friction stir welding enables high-quality welding of aluminum alloys, the welding tool remains a significant challenge that significantly limits its application. Advances in tool material and design are needed to improve affordability and robustness and to expand the processing window. In addition, tool wear is a critical issue. Despite recent progress in this area, further investigation is required into the wear mechanisms and impact of tool debris on the performance characteristics of the processed material.

### 2.2. Friction Welding

**Figure 4:**
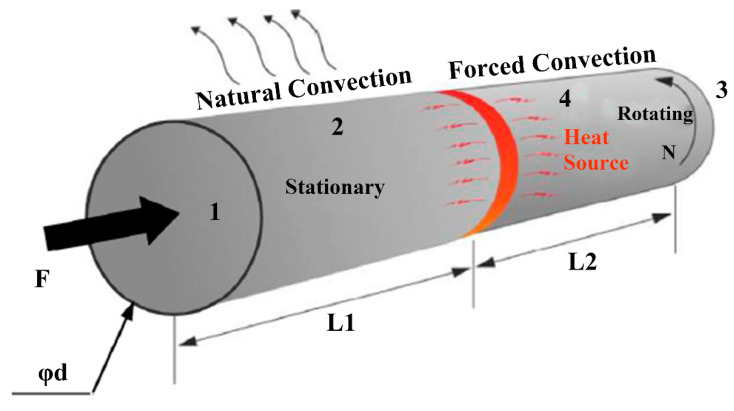
Schematic diagram of the friction welding process [85].

Friction welding is a solid-state joining process that consists of the microscopic joining of the contact surfaces of the parts at temperatures below their melting points. The joining mechanism of friction welding is friction, plastic deformation, extrusion, and recrystallization [79,80,81]. As shown in Figure 4, heat is generated by friction between the two surfaces, which causes the contact surfaces to become thermoplastic. The parts are then driven toward each other with sufficient force to form a metallurgical bond [82,83,84]. Friction welding is conducive to avoiding the process defects that tend to occur in fusion welding. Its joints have the advantage of high mechanical properties. However, friction welding depends on the workpiece rotation, and it is applicable to bar, tubes, and other rotating body welding scenarios; welding non-circular cross-sections is more difficult.

**Figure 5:**
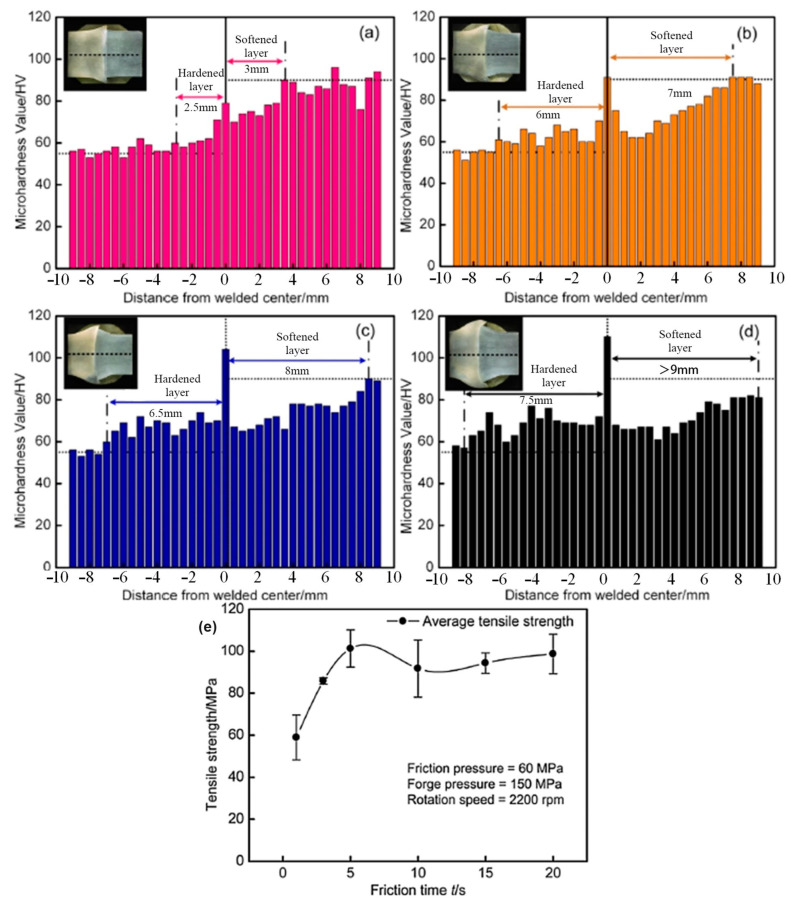
Microhardness distribution along the central line in the Al/Mg welded joints at different times [83]: (**a**) 1 s; (**b**) 3 s; (**c**) 5 s; (**d**) 10 s; (**e**) effects of the friction time on the tensile strength of Al/Mg welded joints.

The rotation speed plays an important role in the microhardness distribution of the joints. Higher rotation speeds and a longer burn of length result in higher tensile strengths [86,87]. For AA6061 aluminum alloy, the local tensile strength at the same location increases and then decreases with the increase in rotation speed. At the same time, the microhardness of the joint also changes with the increase in rotation speed and even shows the opposite distribution at 500 rpm and 1500 rpm [88]. Friction time also determines the mechanical properties of the joint, as shown in Figure 5. Shorter friction times result in lower heat input and make it more challenging for the aluminum alloy to achieve the thermoplastic state [89]. When the reaction between the materials involved is insufficient, non-coalescence cracks can occur [81]. However, the growth of IMC layers was promoted by increasing the friction time, and thicker IMCs will also deteriorate the mechanical properties of the joint. Under this condition, increasing the friction pressure could significantly reduce the IMCs’ thickness of the joint and improve the tensile strength of the joint [83,90]. In addition, the uneven distribution of friction pressure and temperature leads to an uneven distribution of IMCs. An enormous amount of IMCs appeared in the half-radius zone and the periphery zone, but much fewer IMCs appeared in the central zone [90]. To control the thickness of IMCs, in addition to optimizing the welding process [91], it can be achieved by adding a suitable interlayer to the substrate [92].

The grain and second-phase particle evolution of aluminum alloys during friction welding is also heterogeneous and affects the final properties of the joint. High temperatures and severe plastic deformation lead to recrystallization, resulting in significant grain refinement in the friction interface zone [93]. In addition, the larger second-phase particles in the base metal are destroyed as fractures by the frictional force and are thus more uniformly distributed in the weld zone. This contributes to the increase in microhardness in this region [87]. The TMAZ is a mixed microstructure of fine and coarse grains due to insufficient recrystallization caused by low heat input [89], and a significant volume fraction of the second phase is dissolved in the matrix near the streamline [83]. Table 3 lists the summary of the tensile strength of friction welded joints.

### 2.3. Diffusion Welding

**Figure 6:**
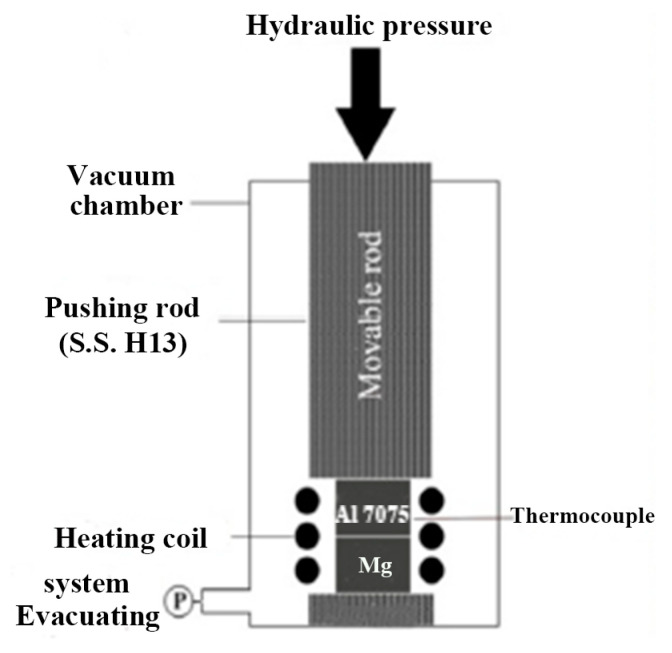
Schematic diagram of the diffusion bonding apparatus [97].

Diffusion bonding is a low-temperature welding process in which both heat and pressure are applied to the joint, resulting in surface micro-deformation, as shown in Figure 6. It is primarily used to join dissimilar metals [96].

**Figure 7:**
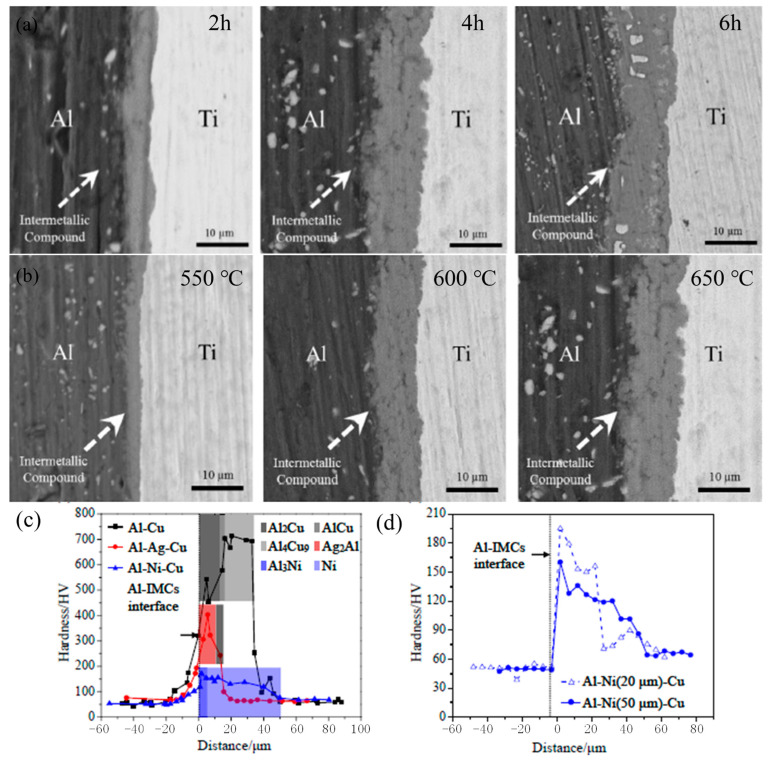
SEM back-scattered electron images showing the interfacial layer of a specimen annealed [96,103] (**a**) for different times and (**b**) at different temperatures. Microhardness profiles (**c**) of the Al-Cu, Al-Ag (10 μm)-Cu, and Al-Ni (50 μm)-Cu joints, of which the bonding parameters are 520 °C/10 MPa/60 min, 460 °C/15 MPa/60 min, and 520 °C/15 MPa/60 min, respectively; microhardness profiles of (**d**) the Al-Ni (20 μm)-Cu and Al-Ni (50 μm)-Cu joints, of which the bonding parameters are 500 °C/10 MPa/60 min.

Higher bonding temperatures and longer bonding times lead to grain coarsening and the formation of brittle IMCs, which reduces joint strength and increases interfacial hardness. Al and Mg alloys mainly form brittle IMCs such as Mg_2_Al_3_ and Mg_17_Al_12_ at the diffusion interface [30,98,99,100], whereas Al and Ti alloys mainly form IMCs such as Al_3_Ti, TiAl, AlTi_3_, and AlCu_2_Ti [101]. The thickness of the IMCs increases with the increase in the bonding temperature and holding time, resulting in a decrease in the joint strength. The plastic collapse of the asperities of the bonding surface leads to intimate contact, which compensates for the embrittlement due to the intermetallic phases. However, as the thickness of the IMCs increases, the resulting embrittlement overbalances the positive effects of the improved coalescence of the faying surfaces. As a consequence, there is a continuous decrease in joint strength and an increase in brittleness [96,102]. As shown in Figure 7a,b, the thickness of IMCs at the joint interface greatly relies on the bonding temperature and time [97]. Higher temperatures lead to thicker IMCs, whereas lower temperatures result in incomplete coalescence of the diffusion surfaces due to the metal’s high flowability. It is worth noting that the yield strength (YS) of the base materials remains high [101].

The main difficulty in diffusion bonding lies in controlling the intermetallic compound of the diffusion interface. The addition of an interlayer is considered a viable method for diffusion bonding. This is because the composition of the interlayer can be flexibly adjusted to meet the requirements of the phase composition and mechanical properties of the joints [104], as shown in Figure 7c,d. Commonly used interlayers include Cu [99], Ni [105], Sn [106], Ag [103], etc. Diffusion bonding of Al-Li alloys can be performed using pure aluminum as an interlayer. The diffusion of alloying elements at the diffusion interface improves the integrity and mechanical properties of the interface. Both the shear strength of the joint and the hardness of the diffusion interlayer are observed to increase considerably with an increase in the bonding temperature.

### 2.4. Superplastic Solid-State Welding

The metal material can easily realize the intimate contact of the solid surface and the diffusion of atoms on both sides of the interface in the superplastic state. Superplastic solid-state welding uses this superplasticity to achieve the joining of the material [107]. Solid-state welding based on superplastic alloys can be classified into superplastic friction welding [108,109], superplastic pressure welding [110,111], and superplastic forming/diffusion bonding (SPF/DB) [112,113].

By sealing the test sheets in a plastic bag filled with argon gas, smaller deformations and shorter holding times can be used to bond Al-alloys in air. The primary factors affecting this process are the nature of the oxide layer on the surface and the stability of the grain structure [114,115]. The bondability of superplastic aluminum alloys in air is also mainly affected by the surface roughness of the sheets. The void status caused by a particular surface roughness would affect the void closure rate, in which atomic diffusion is the dominant mechanism. Therefore, plates with higher surface roughnesses would require longer bonding times [116].

**Figure 8:**
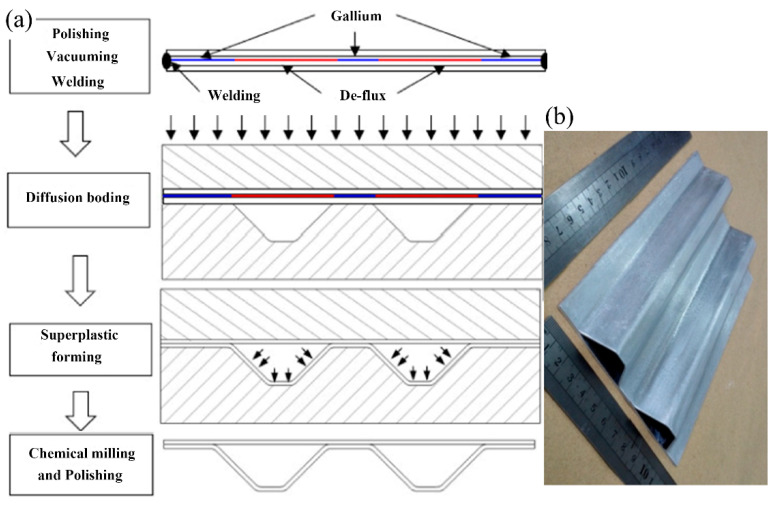
(**a**) Preparation process of the two-layer hollow structure of the 1420 Al-Li alloy. (**b**) two-sheet hollow structure of the 1420 Al-Li alloy [121].

SPF/DB is a technique that utilizes the superplasticity of materials and diffusion bonding to form complex-shaped hollow metal parts or honeycomb structures and is particularly suitable for forming and joining two or more sheets [112,113], as shown in Figure 8. The solid surface oxide film limits the SPF/DB of aluminum alloy. The interlayer diffusion method and the organic coating method [117] enable obtaining reliable joints whose strength and microstructure match the parent material [118]. Additionally, surface roughness impacts SPF/DB [119]. A large surface roughness results in an insufficient contact area at the bonding interface, while a small surface roughness causes microplastic deformation that cannot break the oxide layer [120].

### 2.5. Explosive Welding

**Figure 9:**
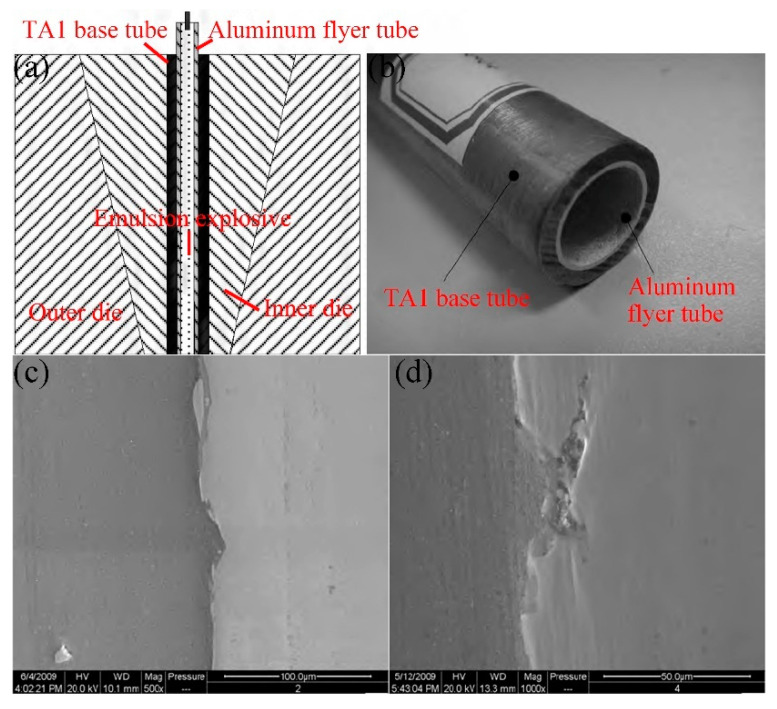
(**a**) Explosive welding device of the clad tube. (**b**) TA1/Al clad tube prepared by explosive welding. (**c**,**d**) Morphology of the TA1/Al clad tube [123].

Explosive welding uses detonation waves to cause high-speed impacts on metal plates, causing localized melting or plastic deformation at the contact surface and producing a tight joint between the materials. It is particularly suitable for joining dissimilar metals and laminated composites [122,123,124], as shown in Figure 9. The joining mechanism at the interface is the result of a combination of pressure welding, diffusion welding, and local fusion welding. The bonding of explosive welding is instantaneous, and the collision speed of the material can reach 300–800 mm/s. Because the atoms at the joint interface do not have enough time to diffuse, the formation of brittle intermetallic compounds is severely limited [125]. The metallic jet and the waveform interface are the main characteristics of the explosive welding interface. The metallic jet depends on the adjustment of the impact velocity and angle, which can break and remove the oxide film on the surface of the sheets, which is conducive to diffusion and metallurgical bonding.

The microhardness of the interface of the explosive welded joint of 7A52 Al alloy and AZ31 Mg alloy is much higher than that of the matrix on both sides. As the distance from the interface increases, the microhardness of the matrix on both sides rapidly decreases to its microhardness range, indicating that there is severe plastic deformation at the interface. However, the work-hardening layer is thin, only a few microns [126]. Because the interface of the explosive welded joint is bonded primarily by mechanical interlocking, the strength of the interface is less than that of friction stir welded or other fusion welded joints. Low explosive mass and high explosive density are advantageous to increase the wavelength and wave amplitude of the interface, reduce the thickness of intermetallic compounds and the number of microcracks [127,128,129], and improve the strength of the welded plates.

### 2.6. Electromagnetic Pulse Welding

**Figure 10:**
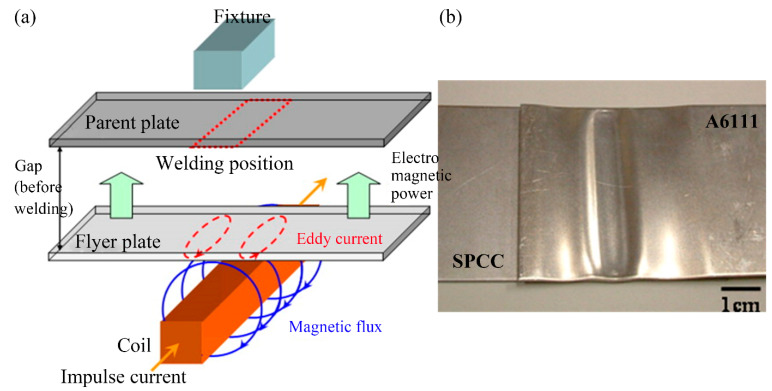
(**a**) Schematic diagram of resistance spot welding with a cover plate. (**b**) SEM images of the weld cross-section at the A5052/SUS304 interface [131].

Electromagnetic pulse welding uses electromagnetic force to drive the welding materials to impact at high speed, promoting surface fracture and material removal at the impact point. This forms a deformed and rough fresh surface, as shown in Figure 10. The two plates are rapidly diffused under local high-temperature and high-pressure conditions to achieve ultra-fast joining [129]. The main forms of EMPW are tube welding and plate welding. EMPW does not produce a heat-affected zone and can greatly reduce the formation of intermetallic compounds at the joint interface [130]. This technique is suitable for joining similar or dissimilar materials. The interface bonding characteristics determine the mechanical properties of the EMPW-processed joint and the optimization design of the welding process window. The wavy morphology is the main interface feature of EMPW. In addition, there are straight interfaces, transition zones, porous structures, and other features [129].

Whether the wavy-like interfacial features appear is directly related to the properties of the weld material itself. The aluminum–aluminum interface mainly shows a wave-like interface, while the aluminum–steel interface and aluminum–copper interface mainly show straight interfaces [33]. The impact velocity increases as the discharge energy increases, and the wavelength and wave amplitude of the interface are determined by the impact velocity between the weld materials [34,131,132]. At the same time, as the discharge energy increases, the thickness of the intermetallic compound at the interface also increases. When the thickness increases to a certain extent, many microcracks and pores are generated [133], which is not conducive to the mechanical properties of the joint.

## 3. Plastic Deformation Strengthening

### 3.1. Physical Surface Modification

**Figure 11:**
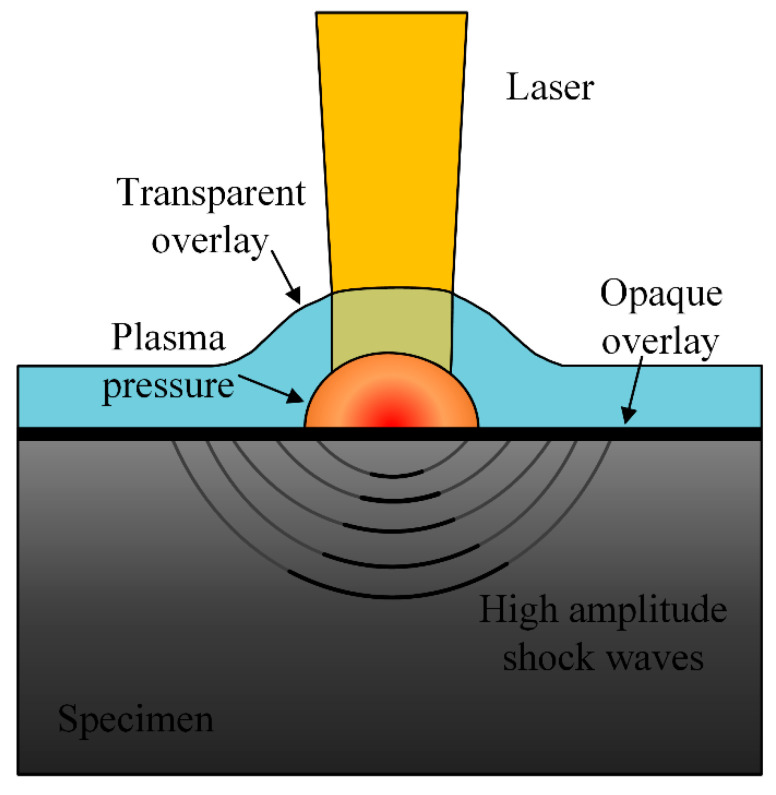
Schematic principle of LSP.

Small deformation physical surface modification, such as mechanical shot peening (MSP), laser shot peening (LSP), and ultrasonic impact treatment (UIT), has been recognized as a promising post-weld treatment method. As shown in Figure 11, the impact of projectiles, shock waves, and other accelerating media on the surface of the workpiece causes the metal to undergo cyclic plastic deformation, thereby hardening the surface and subsurface of the workpiece [134].

Physical surface modification is a cold-working process that produces a large number of non-equilibrium grain boundaries and high-density dislocations on the surface of the workpiece. MSP can improve the microhardness of the surface of the stirred zone of the 6061 aluminum alloy FSW-processed joints by 45 HV and 60 HV at the Almen strengths of 0.18 mmA and 0.24 mmA, respectively [134]. The application of MSP resulted in substantial strain hardening on the crown side, whereby Almen intensities of 0.24 mmA achieved a maximum increase in microhardness of 120 Hv. Conversely, the maximum microhardness on the root side was only 75 HV. Similarly, the microhardness of laser-welded joints was increased from 70 HV to 82.9 HV by LSP. However, it remained lower than the value of 120 HV for BM [135]. Additionally, LSP treatment resulted in a tendency for the surface microhardness of joints to shift from non-uniform distribution to uniform distribution at various locations [136]. Heat treatment based on physical surface modification can further improve the mechanical properties of aluminum alloy welded joints. UIT alone can increase the surface microhardness of 7A52 aluminum alloy welded joints by 42.7%, while the combination of an aging treatment can increase the surface microhardness of joints by 59.1% [137].

**Figure 12:**
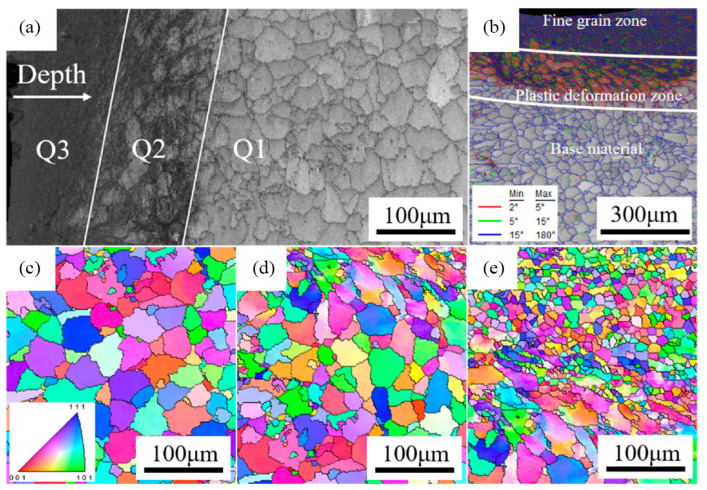
EBSD characterization of laser-welded 7075 Al alloy joint [141]. (**a**) Microstructure zones of the weld zone after aging and ultrasonic impact treatments; (**b**) misorientation angle distribution; (**c**–**e**) Q1-Q3 inverse pole figure.

**Figure 13:**
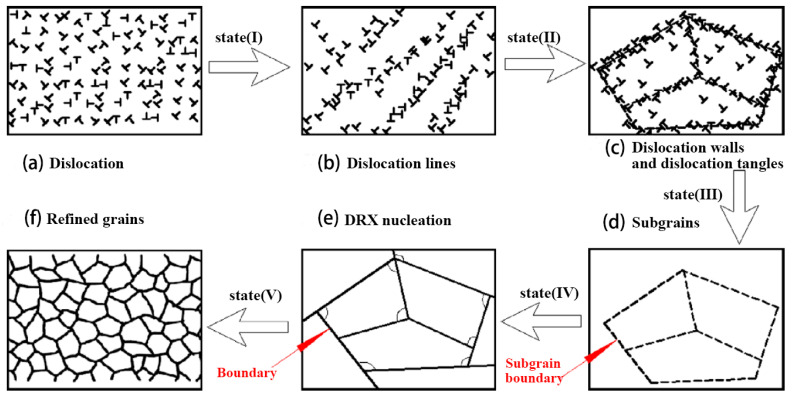
Schematic illustration showing the microstructure evolution process of LY12 Al alloy induced by multiple LSP impacts [139].

However, the effective depth of physical surface modification is very limited, which is mainly limited to the surface layer of the sample, as shown in Figure 12. The maximum effective depth of MSP and UIT amounts to approximately 60 μm [134,137]. Conversely, LSP provides a significantly more profound effective depth, with a potential of up to circa 500 μm [135]. Moreover, the microhardness of the joint exhibits a sharp decline as one moves farther away from the top surface [137,138]. The change in joint microhardness is due to the change in joint microstructure. As shown in Figure 12, the physical surface modification technique can obtain fine grains on the surface of welded joints. As shown in Figure 13, at the initial stage of surface impact treatment, a large number of dislocations are formed inside the original grains. Then, low-angle grain boundaries (LAGBs) are formed through dislocation entanglement, followed by high-angle grain boundaries (HAGBs), which absorb movable dislocations. This culminates in the creation of new, smaller grains [136,139]. The microstructure of the joint surface shows a gradient distribution along the thickness direction, and the grain size increases with increasing distance from the top surface until it reaches the initial size before surface impact treatment [138,140].

Physical surface modification plays a limited role in improving strength but plays a more important role in improving fatigue strength, mainly due to the hardening of the joint surface. For AA6082 aluminum alloy welded joints, shot peening can increase the yield strength and tensile strength of the joints by 10% and 4%, respectively. But it can increase the fatigue strength by 38%, and its fatigue strength is even higher than the average value of the base metal [142]. Due to the impact of projectiles, shock waves, and other accelerating media, many micropores are generated on the joint surface, which cause high compressive residual stresses. In fatigue tests, the high compressive residual stresses on the joint surfaces reduce the tensile residual stresses generated by cyclic loading. This is conducive to reducing the average residual stress in welded joints, inhibiting the formation of fatigue cracks and reducing the crack propagation rate, thus increasing the fatigue life [136,143].

In summary, the physical surface modification technology causes the coarse grains in the surface layer of the joint to be gradually refined to the nanometer scale by strong plastic deformation, and the hardness of the surface layer is greatly improved. At the same time, the residual tensile stress in the surface layer is converted into compressive stress [144] so that the fatigue strength of the joint is greatly improved, as shown in Table 4. However, due to the limited depth of accelerating media action, the improvement in the tensile strength of the joint is not obvious.

### 3.2. Large-Scale Plastic Deformation Technology

**Figure 14:**
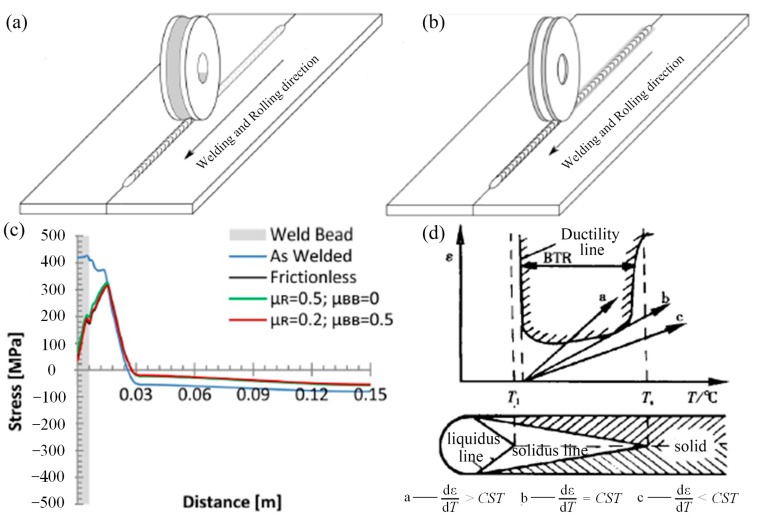
Geometry and rolling methods for [39,151] (**a**) rolling on top of the weld bead and (**b**) rolling beside the weld bead with the dual flat rollers; (**c**) mid-thickness longitudinal residual stress after rolling on top of the weld bead and different friction coefficients between the workpiece and the backing bar (μBB) and the roller (μR); (**d**) schematic diagram of welding hot cracking generation conditions.

The large-scale plastic deformation of joints during the welding process plays a role in controlling weld residual stress and hot cracking [147,148]. The main cause of the buckling deformation of welded plates is the presence of high longitudinal tensile stresses in the weld seam and its vicinity. The application of compressive strain through plastic deformation extends the weld seam and surrounding metal, as illustrated in Figure 14a–c. This reduces the longitudinal residual tensile stresses and minimizes the gap between the average longitudinal residual tensile stresses and the critical buckling stresses, thus controlling the buckling deformation of welded plates [149]. As shown in Figure 14d, the sufficient conditions for the welded joint to generate hot cracking can be represented by Equation (2) [150], where ε is the actual strain in the weld zone or heat-affected zone during the cooling process after welding and T is the temperature. The actual tensile strain of the weld or HAZ, which is in the brittle temperature range, is represented by Equation (3), while Equation (4) gives a criterion for avoiding hot cracking [147]. Table 5 summarizes the large-scale plastic deformation treatments to control weld residual stress and hot cracking. The results show that they are effective at controlling both thermal cracking and residual stress and distortion.

(2) $$\frac{d\varepsilon }{dT}>CST$$

(3) $$\varepsilon^{\prime }=\varepsilon -\varepsilon_{c}$$

(4) $$\frac{d\varepsilon }{dT}-\frac{d\varepsilon_{c}}{dT}<CST$$

**Figure 15:**
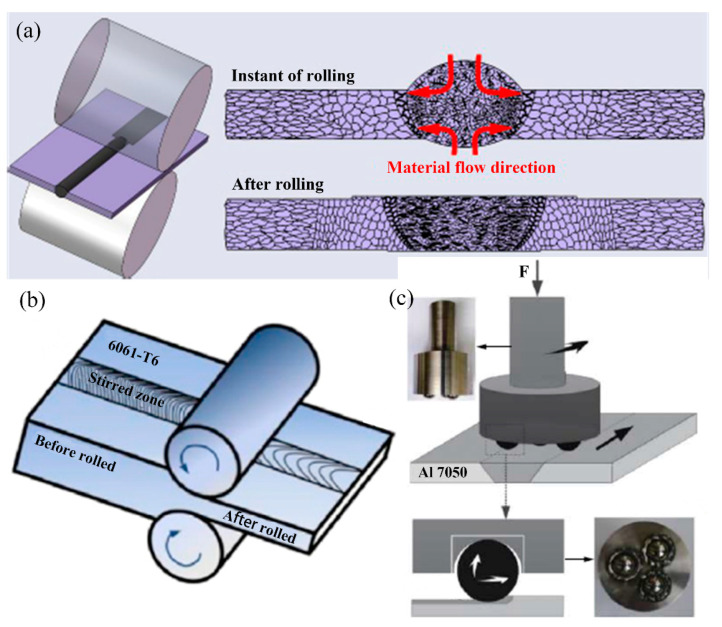
Schematic of the rolling process [158,159,161]. (**a**) Partial rolling; (**b**) Entire rolling; (**c**) Rotation rolling.

Rolling, hammering, and other large-scale plastic deformation techniques can eliminate microstructure defects and significantly refine the grain and second-phase particles [44]. The major rolling treatments are shown in Figure 15. When the specimen is subjected to deformation, the joint properties are mainly improved by the combined effect of work hardening and fine-grain hardening [157]. On the one hand, large plastic deformation refines the grains, increases the grain boundary area, and hinders the dislocation movement to form a dislocation pile-up. On the other hand, during the plastic deformation of the material, dislocation multiplication and intersection take place. The escalation of crystal defects like vacancies, interstitials, and stacking faults amplifies the resistance to dislocation movement, resulting in strain hardening. As a consequence, the microhardness of each region of the joint improves [158,159,160].

**Figure 16:**
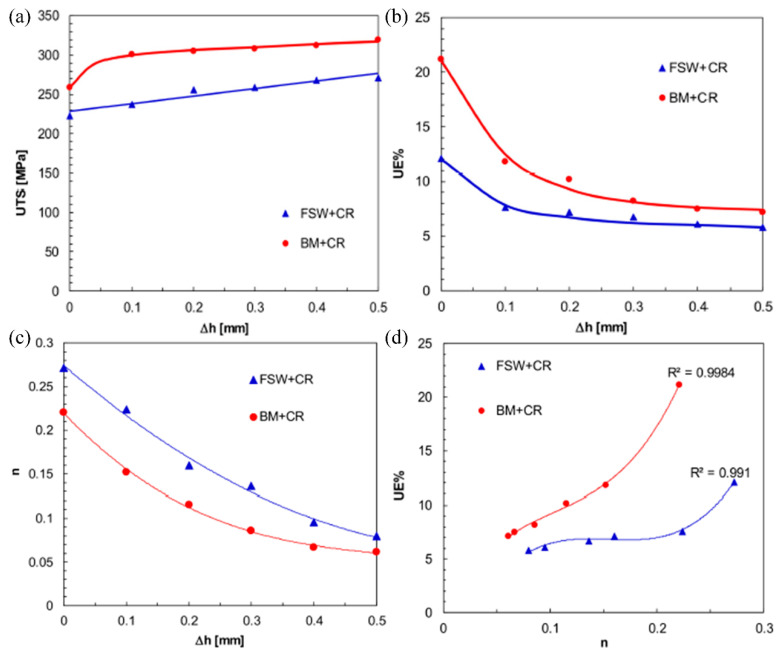
Comparison between the mechanical behavior exhibited by the FSWed + CRed and BM+CRed samples in terms of [162]: (**a**) UTS vs. height reduction; (**b**) elongation vs. height reduction; (**c**) strain hardening exponent vs. height reduction; (**d**) elongation vs. strain hardening exponent.

The degree of work hardening caused by large plastic deformation processes is closely related to the amount of deformation, which is mainly reflected in the strain hardening index and hardening capacity, both of which increase with the increase in the amount of deformation, as shown in Figure 16 [162]. The FSW 6061-T6 plates were rolled as a whole, resulting in a rise in yield strength of the stirred joint zone from an initial 197 MPa to 299 MPa, 331 MPa, and 352 MPa, respectively, when the plates were rolled to 4 mm, 3 mm, and 2 mm. The base material also showed a similar increase in its yield strength [162]. Cold rolling improves the yield strength and tensile strength of aluminum alloy welded joints but also reduces the elongation of the joints. The tensile strength of 6061 aluminum alloy TIG welded joints is 214.4 MPa, and the elongation is 6.4%. When the excess filler metal is cold rolled, the tensile strength of the joints is increased to 254.5 MPa, while the elongation is reduced to 3.1% [161].

**Figure 17:**
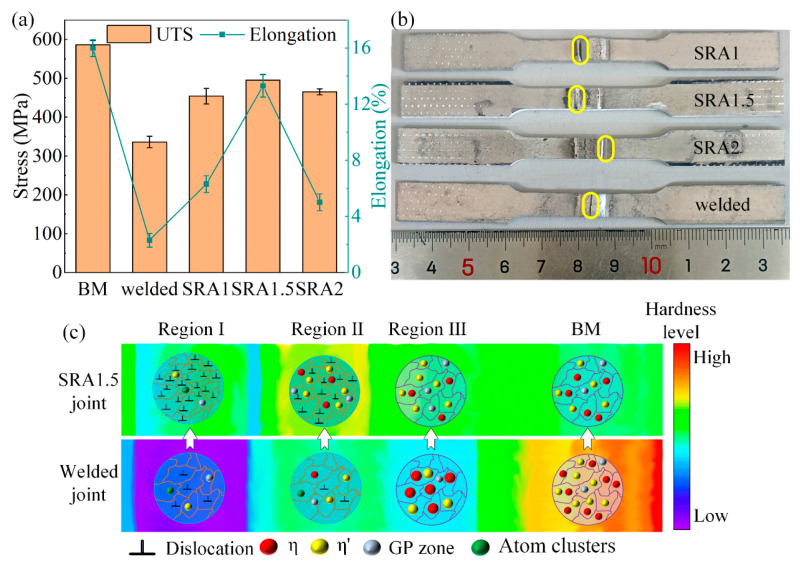
(**a**) Tensile results; (**b**) fracture position (marked by yellow circle); (**c**) strengthening mechanisms of the SRA process [163].

The post-weld composite treatment process, which combines heat treatment and rolling, could simultaneously increase the strength and plasticity of aluminum alloy welded joints. As shown in Figure 17, the “solution treatment-partial rolling-natural aging” (SRA) composite treatment process increased the strength and elongation of 7075-T6 Al alloy welded joints from 336 MPa and 2.3% to 495 MPa and 13.3%, respectively. This is attributed to precipitation hardening and work hardening interacting in different regions of the joints [163]. The “solution treatment-artificial aging-cold rolling” composite treatment process could improve the strength and ductility of 6061-T6 Al alloy welded joints to reach up to 100% and 67% of the base metal, respectively [164]. Compared with the welded joints and the base metal, the hot rolling process increases the Erichsen cupping value of 7075-T6 Al alloy welded joints by a factor of 2.3 and 1.43, respectively. In addition, the tensile strength of the hot-rolled joints can be increased to 476 MPa when subjected to the heat treatment process [157]. The sequence of heat treatment and rolling has a great influence on the variation of the joint properties. Heat treatment after rolling often leads to dislocation recovery and over-aging phenomena, which weakens the work-hardening effect.

Table 6 summarizes the mechanical properties of the aluminum alloy welded joints by large-scale plastic deformation technology. As can be observed, the plasticity of the joint is greatly reduced when the entire joint is rolled, whereas rolling only the weld beam enhances the plasticity. Furthermore, this enhancement in plasticity is even greater after undergoing heat treatment.

**Figure 18:**
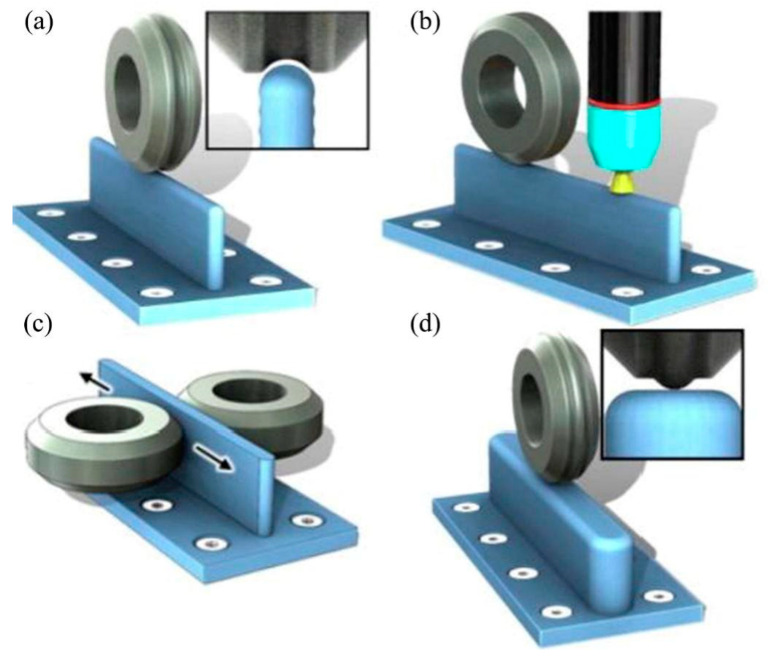
Schematic diagram of the main rolling methods [170]: (**a**) Vertical with a profiled roller; (**b**) in situ rolling; (**c**) pinch rolling; (**d**) rolling with an inverted profiled roller for thick sections and intersections.

Large plastic deformation can also be applied to the additive manufacturing (AM). Figure 18 shows the schematic diagram of hybrid deposition and micro-rolling (HDMR) technology. As shown in Figure 18a,c,d, the cold rolling process can be carried out immediately after the deposition of each layer. As shown in Figure 18b, metal parts can also be produced by hot rolling (the temperature exceeds its recrystallization temperature) immediately after deposition. The micro-roller is mounted close behind the energy source, and as the energy source moves forward, the trailing micro-roller continuously rolls on the top surface of the deposited layer under high temperatures [168]. The accuracy of the HDMR method is significantly improved compared with the freeform deposition manufacturing method. The microstructure of the hybrid manufacturing part becomes cellular crystals instead of dendrites. In addition, the mechanical properties of metal parts are significantly improved by the HDMR method compared with casting, forging, and freedom deposition. This increase is observed in the strength and elongation of the AA5A56 aluminum alloy, which advances from 307.8 MPa and 10.3% for free deposition to 348.7 MPa and 20.4% for the HDMR method [169]. Table 7 lists the tensile properties of aluminum alloys for the as-deposited and HDMR cases.

In summary, the reasons for large-scale plastic deformation technology to improve the performance of aluminum alloy welded joints can be attributed to several aspects. First, plastic deformation can increase the compactness of the microstructure and reduce the number of weld pores. Second, the dislocations that are greatly increased intersect and shear with each other and are pinned by solute atoms and precipitated phase particles, which increases the resistance of dislocation movement and realizes work hardening but is also accompanied by a decrease in plasticity. Third, severe deformation or deformation at high temperatures will cause fine-grain hardening. The combination of plastic deformation and heat treatment can simultaneously improve joint strength and plasticity through strengthening mechanisms such as work hardening, fine-grain hardening, and precipitation hardening. Hybrid welding and micro-rolling technology or hybrid deposition and micro-rolling technology can play a role in controlling residual stresses and welding hot cracking in addition to improving the mechanical properties of components.

## 4. Summary

Aluminum alloys are widely used in many areas of manufacturing, and as a result, numerous studies have been conducted on the welding of Al alloys. The key issues for Al alloy welding are the softening characteristics and the formation of brittle intermetallic compounds, which adversely affect the joint performance. The welding/plastic deformation hybrid technology is an important way to improve the performance of Al alloy welded joints. Detailed research progress and results have been reported by many researchers.

Plastic deformation welding can realize the high-quality joint of aluminum alloy with the same or dissimilar materials and can avoid the common defects in fusion welded joints. However, the processing parameters have a great influence on the quality of the joint. First, it affects the mixing of materials by influencing the heat input. Second, it affects the quality of the joint by affecting the thickness and distribution of the IMC for dissimilar materials. Some externally assisted technologies can further improve the performance of the joint, such as forced cooling, ultrasonic assistance, and the addition of interlayers.

Plastic deformation in the weld zone improves the microstructure and mechanical properties of the joint by introducing dislocations, sub-structures, and surface stresses. Physical surface modification is beneficial for increasing the surface hardness of the weldment, and the surface hardness of the joint can be further increased when combined with heat treatment. This method contributes little to the strength improvement, but it can effectively improve the fatigue strength of the joint. The disadvantages are limited effective depth of physical surface modification and gradient hardness distribution. The hardening degree of large-scale plastic deformation to the welded joint is related to the deformation amount, and the plasticity of the joint will be reduced while improving the strength. The composite process of plastic deformation and heat treatment can synergistically improve the strength and plasticity of the joint. In addition, the use of large-scale plastic deformation treatment during the welding process can improve the residual stress distribution of the joint and play a role in preventing welding hot cracking.

## 5. Conclusions

At present, research on plastic deformation strengthening has been sufficient, but there are still many problems to be solved. First, there is a clear need for more efforts to achieve a synergistic improvement in the strength and plasticity of aluminum alloy welded joints rather than simply increasing joint strength. Then, further systematic studies need to be carried out to understand the evolution of the microstructure and mechanical properties under the thermomechanical coupling conditions in order to avoid the negative effects associated with cold deformation condition, such as cracking, plasticity degradation, and low performance enhancement. Furthermore, further work is needed to develop an integrated preparation technology for key components of high-strength aluminum alloys based on welding/plastic deformation composite forming technology to achieve a simultaneous completion of welding and strengthening.

## Figures and Tables

**Table 1:** Summary of the tensile properties of the high-strength aluminum alloy butt joints welded by the fusion welding method.

Base Metal	Welding Method	UTS of BM (MPa)	Elongation of BM	UTS of Joint (MPa)	Elongation of Joint (%)	Joint Efficiency (Joint/BM, %)	Ref.
AA2024	LBW	463	14.9	364	2.4	78.6	[11]
AA2A12	CMT	300	16	220	10.0	73.3	[12]
AA2060	LBW	495	13.9	304.4	7.6	61.5	[13]
AA2519	MIG	479	11.2	275	3.0	57.4	[14]
AA6022	LBW	233	28.5	170	2.5	72.9	[15]
AA6N01	CMT	309	12.6	215	10.6	69.6	[16]
AA6082	MIG	310	6.0	178	4.7	57.4	[17]
AA7075	TIG	578	12.2	300	1.8	51.9	[18]
AA7075	LBW	495	16.2	328	2.5	66.3	[19]
AA7N01	MIG	406	16.3	289	4.7	71.2	[20]
AA7A52	Laser-MIG	475	14.4	317	3.7	66.7	[21]

**Table 2:** Summary of the tensile properties of FSW aluminum alloy butt joints.

Materials	UTS of BM (MPa)	El of BM (%)	UTS of Joint (MPa)	El of Joint (%)	Joint Efficiency (%)	Ref.
AA2A14	469	9.7	344	9.1	73.3	[57]
AA2219	435	5.3	315	3.3	72.4	[63]
AA5083	304	23.1	303	19.0	99.6	[64]
AA6061	331	11.7	237	5.2	71.6	[65]
AA6082	293	8.1	195	5.9	66.6	[66]
AA7075	556	18.2	445	7.6	80.0	[67]

**Table 3:** Summary of the tensile strength of friction welded joints.

Materials	FW Method	UTS of BM (MPa)	UTS of Joint (MPa)	Joint Efficiency (%)	Ref.
AA2024	Continuous drive FW	502	462	92.0	[93]
AA2139	Linear FW	487	452	92.8	[94]
AA2024	Continuous drive FW	450	390	86.7	[95]
AA6061	Rotary FW	335	295	88.1	[88]
Semi-solid metal 7075	Rotary FW	/	105	/	[87]
AA5A33/AZ31B	Continuous drive FW	352/271	101	37.3	[83]

**Table 4:** Summary of the fatigue properties of aluminum alloy welded joints treated by physical surface modification.

Materials	Welding Method	Physical Surface Modification	Test Conditions	Fatigue Properties of Welded Joint	Fatigue Properties of Treated Joint	Ref.
AA5083	TIG	Shot peening	10^5^ cycles	49 MPa	78 MPa	[145]
			10^6^ cycles	28 MPa	54 MPa	
AA6061-T6	TIG	Warm laser peening	172 MPa	43,703 cycles	75,683 cycles	[143]
			120 MPa	87,850 cycles	120,809 cycles	
AA6082.5-T6	MIG	Shot peening	10^6^ cycles	100 MPa	138 MPa	[142]
AA7050-T7451	FSW	Shock peening	200 MPa	10 × 10^5^ cycles	13 × 10^5^ cycles	[136]
AA7075	LBW	Ultrasonic impact	2 × 10^6^ cycles	48.6 MPa	103.0 MPa	[146]

**Table 5:** Summary of the large-scale plastic deformation treatments to control weld residual stress and hot cracking.

Deformation Technique	Purpose	Materials	Welding Method	Deformation Location	Ref.
Synchronous rolling during welding	Prevent hot cracks and improve mechanical performance	LY12CZ	TIG	Both sides of the weld, the weld beam	[147,152]
Synchronous rolling during welding	Prevent hot cracks	LY12CZ	Melt coating	Both sides of the weld	[150]
Synchronous rolling during welding	Prevent hot cracks	AA2024-T4	TIG	Both sides of the weld	[153]
Welding with trailing impactive rolling	Control residual stress and hot cracks	LY12CZ	TIG	Weld bead and weld toe	[148]
Welding with rotating extrusion	Reduce residual distortion	AA2A12-T4	TIG	Weld beam	[149]
Welding with trailing peening	Prevent hot cracks	AA2A12	TIG	Both sides of the weld	[39]
Welding with hammering and rolling	Prevent hot cracks and welding distortion	LY12CZ	TIG	Weld beam	[154]
Laser roll welding	Produce Al/Steel dissimilar joints	AA5052 and SPCC steel	LBW	Overlap area	[154]
Laser roll welding	Produce Al/Ti dissimilar joints	AA5052 and H4600 Ti alloy	LBW	Overlap area	[155]
Welding with rolling	Prevent distortion	AMg6 Al alloy	TIG	Weld beam	[156]

**Table 6:** Summary of the mechanical properties of the Al alloy welded joints by large-scale plastic deformation technology.

Deformation Technique	Deformation Location	Materials	Welding Method	UTS of Welded Joints	UTS of Treated Joints	El of Welded Joints	El of Treated Joints	Ref.
Cold rolling	The whole joint	AA5754	FSW	220	270	12%	5.8%	[162]
Cold rolling	The whole joint	AA5754	FSW	225	244	/	/	[165]
Cold rolling	The whole joint	AA6061	FSW	229	369	20%	6%	[158]
Cold rolling	Weld beam	AA7075	Laser-TIG	365	454	3.3%	6.6%	[44]
Hot rolling then heat treatment	Weld beam	AA7075	TIG	336	479	2.3%	3.9%	[157]
Cold rolling and heat treatment	Weld beam	AA7075	TIG	336	495	2.3%	13.3%	[163]
Cold rolling then heat treatment	Weld beam	AA6061	TIG	214	305	6.4%	9.8%	[164]
				**Fatigue life of welded joints (cycles)**		**Fatigue life of treated joints (cycles)**		
Rotation rolling	Weld beam	AA7050	FSW	3.6 × 10^5^		2.2 × 10^6^		[159]
Heat treatment then deep rolling	The whole joint	AA7075	FSW	23,846		56,968		[166]
High frequency impacting and rolling	The whole joint	AA2A12	Plasma arc	15,799		58,436		[167]

**Table 7:** Tensile properties of aluminum alloys for the as-deposited and HDMR cases (where V represents vertical direction; H represents horizontal direction).

Materials	Tensile Direction	UTS of Deposited (MPa)	YS of Deposited (MPa)	El of Deposited (%)	UTS of HDMR (MPa)	YS of HDMR (MPa)	El of HDMR (%)	Ref.
AA2319	V	314	244	6.2	262	130	15.5	[171]
	H	325	250	8.5	263	135	18.6	
Al-4.7 Si	V	134	52	12.3	159	72	16.2	[172]
AA2319	V	267.8	109.4	14.5	293	119.9	/	[173]
	H	296	111.3	23.0	324	122	/	
Al-Cu6.3	V	260	/	/	313.6	/	/	[174]
Al-Mg4.5	V	290	/	/	342.8	/	/	
AA2024	H	324	204	7.7	394	308	7.3	[175]
	V	267	186	2.4	280	273	0.5	
AA5087	H	291	142	22.4	344	240	20.1	[176]
